# The challenge of neuropsychiatric manifestations in parkinson’s disease. A case report

**DOI:** 10.1192/j.eurpsy.2021.671

**Published:** 2021-08-13

**Authors:** A. Cerame Del Campo, P. Coucheiro Limeres, A. Franco Soler

**Affiliations:** Centro De Salud Mental, Instituto Psiquiátrico José Germain, Leganes, Spain

**Keywords:** parkinson’s disease, management, neuropsychiatric manifestations

## Abstract

**Introduction:**

We present the case of an 82-year-old patient who was treated by our liaison psychiatry unit after a suicide attempt through prescription-drug overdose. The patient had been diagnosed with Parkinson’s disease (PD) ten years prior to his admittance and was being treated with carbidopa/levodopa and non-ergot dopamine agonists.

**Objectives:**

Impulse control disorders and depression are the most prevalent neuropsychiatric manifestation of PD. According to several sources, this symptomatology is underdiagnosed and undertreated, causing helplessness and distress to patients and their caregivers. Likewise, the accumulated evidence suggests that certain drugs can contribute to the appearance of the aforementioned symptoms.

**Methods:**

A case report is presented alongside a review of the relevant literature regarding the neuropsychiatric manifestations in the context of PD and the diagnosis and treatment of these symptoms.

**Results:**

During his treatment, ropinirole was removed while quetiapine was progressively administered (up to 150mg/day). Carbidopa/levodopa regime was increased causing visual hallucinations and delusional jealousy. A careful balance between antiparkinsonian and antipsychotic medication needed to be achieved before discharge.

**Conclusions:**

Neuropsychiatric manifestations in the context of PD are more prevalent than what was thought in the past. Certain medications, particularly non-ergot dopamine agonists could potentially contribute to the onset of these symptoms. Moreover, these manifestations can be underdiagnosed due to the stigma or social burden imposed upon family and / or caregivers. It is important that recent advances in the understanding of non-motor symptomatology of PD could permeate clinical practice to achieve an adequate identification and treatment of these symptoms.

